# Cirrhotics with Monocyte Chemotactic Protein 1 Polymorphism are at Higher Risk for Developing Spontaneous Bacterial Peritonitis – A Cohort Study

**Published:** 2021-05-27

**Authors:** K. Vamsi Murthy, Meenu Subrahmanian, Thiagarajan Sairam, Venkatakrishnan Leelakrishnan, Ramalingam Sankaran

**Affiliations:** ^1^Department of Gastroenterology, PSG Hospitals, Coimbatore, Tamil Nadu, India; ^2^PSG Center for Molecular Medicine and Therapeutics, PSG Institute of Medical Sciences and Research, Coimbatore, Tamil Nadu, India; ^3^Academic Research Consultant (Molecular Biology), Coimbatore, Tamil Nadu, India

**Keywords:** monocyte chemotactic protein 1, polymorphism, spontaneous bacterial peritonitis, liver cirrhosis, ascites

## Abstract

**Background:**

Spontaneous bacterial peritonitis (SBP) is a complication of liver cirrhosis and its occurrence portends poor patient survival. There is emerging evidence that genetic predisposition could significantly alter the occurrence and course of SBP. Monocyte chemotactic protein 1 (MCP1) is a potent chemokine that perpetuates the pro-inflammatory milieu in SBP.

**Aim:**

This study aimed at investigating *MCP1* genotype polymorphism and its survival impact in patients with decompensated liver cirrhosis.

**Methods:**

We recruited 107 individuals with decompensated liver cirrhosis and categorized them into two groups. Patients having SBP formed the cases (Group 1) and controls were patients without SBP (Group 2). *MCP1* polymorphism (−2518A/G) was assessed in both groups by restriction fragment length polymorphism method. The Chi-square test was used to assess the differences in categorical variables and Kaplan–Meier analyses were used to assess the survival.

**Results:**

Patients with SBP (36.5%) had higher frequency of G allele than patients without SBP (23%) (P=0.031; odds ratio=1.955, 95% confidence interval: 1.0553-3.6216). Kaplan–Meir analysis revealed that presence of SBP (P=0.030) and G allele (P=0.021) had significantly reduced the likelihood of survival among cirrhotics.

**Conclusions:**

Cirrhotic patients with *MCP1* G allele have a higher risk for developing SBP. In general, the presence of the *MCP1* polymorphic G allele (AG/GG genotype) reduced the likelihood of survival among patients with cirrhosis.

**Relevance for Patients:**

This study identifies a critical subgroup of patients with SBP and also predicts prognosis in these individuals. The presence of this genetic polymorphism in addition to the underlying clinical condition may prompt aggressive monitoring, treatment, and follow-up.

## 1. Introduction

The term, spontaneous bacterial peritonitis (SBP) was first coined by Correia and Conn in 1975 [1]. Since then, multiple insights into understand the pathophysiological mechanisms have been identified. The most agreed hypothesis for the development of this entity is bacterial translocation. A high peritoneal neutrophil count (>250 cells/mm^3^) and/or positive culture are important features of this condition. Even with prompt diagnosis, management, and patient care, this condition has a high mortality of 15-30% [2]. Hence, there is a need for newer insights to this condition. Recently, an emerging role of genetic polymorphisms (MCP1, NOD2, and TLR2) and their association with the occurrence of SBP [3] was reported.

Monocyte chemotactic protein 1 (MCP1) is a key chemokine for monocytes, macrophages, and lymphocytes [4] and affects neutrophil infiltration [5,6]. Mutations in the distal regulatory region of *MCP1* gene (at position −2518) were identified [7] and were shown to predispose to SBP. The present study was undertaken to evaluate *MCP1* promoter polymorphism −2518A/G (rs1024611) as a genetic risk factor for SBP in Indian cirrhotic patient cohort.

## 2. Materials and Methods

### 2.1. Study group

In this prospective study, we recruited 107 patients of decompensated liver cirrhosis with ascites who attended the Department of Gastroenterology, PSG Hospitals, Coimbatore, Tamil Nadu, India. Cirrhosis was diagnosed by the clinical, laboratory, and radiological findings. Criteria for inclusion were liver cirrhosis and ascites detected by abdominal ultrasound. Criteria for exclusion were pre-existing chronic renal failure requiring hemodialysis, pre-existing heart failure (New York Heart Association stage III/IV), advanced hepatocellular carcinoma (Barcelona clinic liver cancer-Stage C or greater), malignant ascites, and secondary bacterial peritonitis. The recruitment of patients was approved by Institutional Human Ethical Committee, PSG Institute of Medical Sciences and Research, Coimbatore, and was in accordance with the Helsinki Declaration. The work was commenced after obtaining written informed consent.

Diagnostic paracentesis was performed under strict aseptic precautions in all patients. When minimal ascites was present, ultrasound-guided paracentesis was preferred. Aspirated ascitic fluid was analyzed for polymorphonuclear (PMN) count, albumin, protein, and cytology. Simultaneous blood and ascitic fluid cultures were sent for assessing microbial growth. Diagnosis of SBP was made according to standard protocols [8] (presence of PMN count >250 cells/mm^3^ and^/^or positive bacterial culture from ascites). Patients were divided into two groups. Group 1 consisted of decompensated liver cirrhotic patients with SBP (*n*=63) and Group 2 consisted of cirrhotic patients without SBP (*n*=44). After recovering from SBP, appropriate antibiotic prophylaxis was given as per current guidelines. All patients were followed until death or till the end of our observation period of 4 months after inclusion.

### 2.2. MCP1 genotyping

Whole blood was collected in EDTA coated vials for genomic DNA isolation using EZ-10 spin column blood genomic DNA purification kit (Bio Basic Inc., Canada). Using the 100 ng DNA as a template, *MCP1* gene fragment (930 bp) was amplified using MCP-1F: 5’-CCGAGATGTTCCCAGCACAG-3’ and MCP-1R: 5’ CTGCTTTGCTTGTGCCTCTT-3’ primers in a thermal cycler (Eppendorf, USA) and Taq DNA Polymerase 2X Master Mix (Ampliqon, Denmark) under the cycling conditions of 94°C for 50 s, 55°C for 50 s, 72°C for 1 min for 40 cycles, and a final extension of 72°C for 10 min. The PCR products were resolved on 2% agarose gel to confirm the amplification. *MCP1* −2518A/G polymorphism was genotyped by restriction fragment length polymorphism analysis using 10U of *PVU*II (NEB, USA) enzyme to digest the PCR fragment in 10X buffer at 37°C for 1 h and separation on 12% polyacrylamide gel. The presence of GG genotype yields two bands at 708 and 222 bp. Samples showing undigested 930 bp fragment were the AA genotypes while samples with three bands at 930, 708, and 222 bp were typed as AG.

### 2.3. Statistical analysis

All the statistical calculations were done through GraphPad Prism 5.0. The data are reported as mean of individual figures or frequency (%). Chi-square test was used to assess differences in categorical variables between groups and Student’s *t-*test was performed for comparing the means between two groups. Odds ratio was calculated to evaluate the association of *MCP1* genotype with SBP. Kaplan–Meier curves were used to assess the survival of variables. Comparisons of overall survival were performed using a Log-rank (Mantel-Cox) test. Cox proportional hazard model analysis was carried out to analyze the survival curves using SPSS software (version 24). *P*<0.05 using a two-tailed test was considered significant for all statistical tests.

## 3. Results

### 3.1. Characteristics of the study population

The current study included 107 individuals with decompensated liver cirrhosis. Group 1 was patients with SBP (*n*=63) and Group 2 was patients without SBP (*n*=44). The two groups did not differ in their baseline characteristics such as age, gender, and etiology for liver cirrhosis. The characteristics of the study population are depicted in Table 1.

**Table 1:** Clinical characteristics of patients with liver cirrhosis and ascites in present study

Characteristics of patients	Group 1 (individuals with SBP)(*n*=63)	Group 2 (individuals without SBP)(*n*=44)
Mean age, years (range) †	53 (25-82)	54 (19-83)
Etiology of liver cirrhosis, *n* (%)†		
Alcohol	34 (54)	20 (45)
HBV infection	4 (6)	2 (4.5)
HCV infection	9 (14)	4 (9)
NAFLD	9 (14)	9 (20)
Cryptogenic	5 (8)	7 (16)
Autoimmune	2 (3)	-
Wilson’s disease	1(1.5)	2 (4.5)
Laboratory parameters		
Bilirubin (mg/dl)†	7.6	10
PT/INR*	2.6	2.0
Sodium (mEq/L)†	132	133
Creatinine (mg/dl)†	1.6	1.5
Albumin (g/dl)†	2.3	2.4
Ascitic cell count (cells/ml)*	7300	50
Prognostic indices		
Child–Pugh class, n (%)†		
B	16 (25)	15 (34)
C	47 (75)	29 (66)
Child score†	11	10
MELD score†	25	23
MELD Na†	26	23

Results are expressed as mean of individual figures or frequency (%) as required.

* *P*=statistically significant (*P*<0.05), †*P*=statistically non-significant

### 3.2. MCP1 genetic polymorphism and SBP

The *MCP1* polymorphism (−2518A/G) was evaluated in both groups. The genotype distribution in the two groups was in agreement with the Hardy–Weinberg equilibrium (*P*=0.8 for Group 1 and *P*=0.28 for Group 2). Patients with SBP had higher frequency of G allele (36.5%) and this was statistically significant (*P*=0.031; odds ratio [OR]=1.955, 95% confidence interval [CI]: 1.0553-3.6216) (Table 2). The frequencies of genotypes, AA, AG, and GG were found to be statistically insignificant among the two groups (*P*=0.08). Considering the dominant effect of the G allele, we have applied the additive model with AG/GG genotype and compared it with the AA genotype to ascertain the influence of this polymorphism on disease progression. The frequency of AG/GG variant was significantly higher in individuals with SBP (60%) than without SBP (39%) (*P*=0.02; OR=2.41, 95% CI: 1.0964-5.3156) (Table 3). A higher odds ratio indicated that the population with the AG/GG genotype was at a higher risk of developing SBP. Furthermore, patients with AG/GG genotype also had a high yield of positive ascitic fluid culture positivity (20%) than with the AA genotype (7.7%) (Table 4). Gram-negative organisms were predominant in both the groups and Gram-positive organisms were found only in patients with AG/GG genotype in this study.

**Table 2:** *MCP1* allele frequency in the study population

Allele	Cirrhotics with SBP (*n*=63)	Cirrhotics without SBP (*n*=44)	*P* value	OR
A	80 (63.5)	68 (77)	0.031	1.955 (95% CI: 1.0553-3.6216)
G	46 (36.5)	20 (23)		

Results were expressed as n (%) for allele distribution. Chi-square test was significant for allele frequency between cirrhotics with SBP versus cirrhotics without SBP, OR=Odds ratio

**Table 3:** Additive model of genotype comparison in the study population

Genotype	Cirrhotics with SBP (*n*=63)	Cirrhotics without SBP (*n*=44)	*P* value	OR
AA	25 (40)	27 (61)	0.02	2.41
AG/GG	38 (60)	17 (39)		(95% CI: 1.0964-5.3156)

Results were expressed as *n* (%) for all groups. Chi-square test was significant for genotypes between cirrhotics with SBP versus cirrhotics with non-infected ascites, OR=Odds ratio

**Table 4:** Organisms found in ascitic fluid culture

Organisms	AG/GG genotype (*n*=55)	AA genotype (*n*=52)
*Escherichia coli*	5	2
*Klebsiella pneumoniae*	3	1
*Streptococcus pneumoniae*	1	0
Coagulase-negative staphylococci	2	0
*Brevundimonas vesicularis*	0	1
Total, *n* (%)	11 (20)	4 (7.7%)

### 3.3. MCP1 variants have poor survival

**Figure 1:**
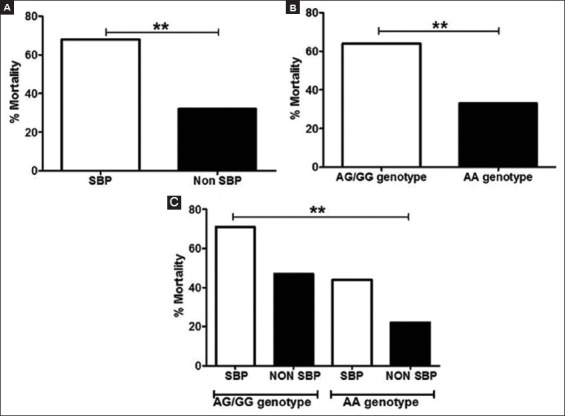
Percentage mortality in the present study. (A) Mortality was higher in patients with SBP (68%) than non SBP (32%). Two-tailed Fisher’s exact test was done to evaluate the significance (**P = 0.005). (B) Mortality was higher in individuals with AG/GG genotype (64%) than with AA genotype (33%). Two-tailed Fisher’s exact test was done to evaluate the significance (**P = 0.001). (C) Individuals with AG/GG genotype showed a higher mortality rate (71% in SBP patients and 47% in non SBP patients) than with AA genotype (44% in SBP patients and 22% in non SBP patients). Chi-square test was done to evaluate the significance between values (**P = 0.001)

In total, 52 patients (48.59%) died. Table 5 summarizes the causes of death for these patients, with acute-on-chronic liver failure representing the major cause. Cirrhotic patients with SBP (Group 1) in general had a significantly higher percentage of mortality (68%) than non-SBP group (32%, *P*=0.005, Figure 1A). Consistently, all patients (both Group 1 and Group 2) with AG/GG genotype had a significantly higher mortality rate (64%) when compared with AA genotype (33%, *P*=0.001, Figure 1B). Individuals with AG/GG genotype and SBP had poor survival and higher mortality rate compared to those without SBP (71% for Group 1 and 47% for Group 2). Whereas, cirrhotic patients in the absence of G allele (AA genotype) had 44% of mortality rate in SBP and 22% in the non-SBP group (*P*=0.001, Figure 1C).

**Table 5:** Analysis of causes of mortality among cirrhotics with different genotypes

Causes of mortality *n* (%)	Dead cirrhotic patients (*n*=52)	

	AG/GG genotype (*n*=35)	AA genotype (*n*=17)
Acute-on-chronic liver failure*	29 (83)	10 (59)
Hepatorenal syndrome	2 (5.7)	1 (5.8)
Gastrointestinal hemorrhage	1 (2.8)	2 (11.7)
infection/SIRS	1 (2.8)	2 (11.7)
Cardiovascular and respiratory causes	1 (2.8)	1 (5.8)
Not specified	1 (2.8)	1 (5.8)

Fisher’s exact test was performed,

* *P*=0.0005. For other parameters, the results were nonsignificant

**Figure 2:**
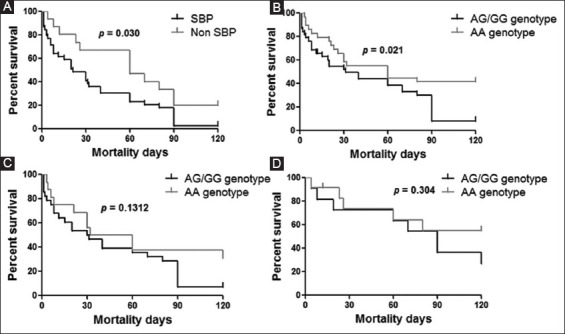
Kaplan-Meier survival curve for overall survival of cirrhotics. (A) With and without SBP, (B) With different genotypes of MCP1, (C) Among SBP group with AG/GG and AA genotypes of MCP1 and (D) Among non SBP group with AG/GG and AA genotypes of MCP1. For both (A) & B), the curves indicate a statistically significant reduction in overall survival due the presence of SBP (*P* = 0.030) and presence of AG/GG genotype (*P* = 0.021). (C) Among cirrhotics with SBP, AG/GG variants showed a statistically insignificant reduction in survival than AA genotype (*P* = 0.1312) & (D) Among cirrhotics without SBP, AG/GG variants showed a reduction in survival than AA genotype which was not significant (*P* = 0.304). Log-rank test (Mantel-Cox) test was utilized for comparisons of survival curves. The survival curves corrected for the presence of hepatocellular carcinoma and Child-Pugh class were represented.

All individuals in the present study were followed up for 120 days. Kaplan–Meier survival curve analysis was carried out (Figure 2) after making corrections for hepatocellular carcinoma and Child–Pugh class. Overall, Group 1 showed a significantly lower survival than Group 2 with a hazard ratio of 2.007 (95% CI=1.067-3.775, *P*=0.030; Figure 2A). Cirrhotic patients with G allele (AG/GG genotype) had a poor survival than those without G allele (AA genotype), which was statistically significant with a hazard ratio of 1.967 (95% CI=1.107-3.497, *P*=0.021; Figure 2B). The presence of G allele resulted in statistically insignificant reduction in overall survival of cirrhotic patients with SBP (hazard ratio of 1.708, 95% CI=0.8523–3.423, *P*=0.1312; Figure 2C) and cirrhotic patients without SBP (hazard ratio of 1.787, 95% CI=0.590-5.409, *P*=0.304; Figure 2D) compared with patients having AA genotype.

## 4. Discussion

SBP is an infection in ascitic fluid without an evident intra-abdominal surgically treatable source. A positive ascitic fluid bacterial culture and/or an elevated ascitic fluid absolute PMN leukocyte (PMN) count (≥250 cells/mm^3^) [8] confirm its diagnosis. In spite of optimal therapy, the occurrence of SBP portends high mortality (15-30%) [2]. Individuals with SBP had a significantly higher percentage of mortality (68%) than those without SBP (32%, *P*=0.005, Figure 1A). This study also reveals SBP as an independent predictor of mortality in cirrhotics.

MCP1 is a key chemokine that increases the influx of pro-inflammatory monocytes, macrophages, activated lymphocytes, and natural killer cells into the ascitic fluid in SBP patients [9]. These cells further perpetuate the pro-inflammatory milieu by secreting cytokines. *MCP1* polymorphism has been studied to play role in various infectious and inflammatory conditions [10-14]. Its role in mediating inflammatory response in chronic liver diseases has been well substantiated [15,16]. Its gene expression has further shown to decrease after treatment, thus emphasizing its importance in the pathogenesis of SBP [17].

The presence of this G allele was seen to predispose the cirrhotic patients to a progressive disease course [17]. Our findings show an increased frequency of G allele in alcohol-related cirrhosis, which is consistent with that of Gäbele *et al*. [18]. Salama *et al*. [17] showed that AG polymorphism in cirrhotics predisposed to SBP. This finding is in agreement with our study, wherein AG genotype frequency was higher in SBP (47.6% of AG). The same group also reported a higher frequency of GG genotype in cirrhotic patients without SBP. However, our study findings are contrary to this.

## Conclusions

In the present study, poor survival was observed in cirrhotic patients with SBP (hazard ratio=2.007) and having *MCP1* G allele (hazard ratio=1.967) when compared with patients without SBP and G allele, respectively (Figure 2A and B). This observation indicates that *MCP1* polymorphism is an additional factor contributing to mortality in cirrhotic patients. Even patients without SBP having the AG/GG genotype have shown higher mortality rate than those with AA genotype. This emphasizes that AG/GG genotype is an independent and important predictor of mortality even in the absence of SBP. This study identifies a subset of patients who would benefit from aggressive surveillance and management.
